# Neonatal Osteomyelitis Caused by *Staphylococcus aureus*: Case Series and Review of the Literature

**DOI:** 10.3390/children13060780

**Published:** 2026-06-03

**Authors:** Maddalena Comune, Irene Furnari, Erika Silvestro, Simone Spolaore, Federica Percivati, Silvia Nurisso, Silvia Garazzino, Marco Denina

**Affiliations:** 1 Department of Pediatrics and Public Health, University of Turin, Regina Margherita Children’s Hospital, 10126 Turin, Italy; 2 Infectious Diseases Unit, Department of Pediatrics, University of Turin, Regina Margherita Children’s Hospital, 10126 Turin, Italy; 3 Orthopedic Unit, Department of Pediatrics, University of Turin, Regina Margherita Children’s Hospital, 10126 Turin, Italy

**Keywords:** neonatal osteomyelitis, *Staphylococcus aureus*, bone infection, neonatal osteoarthritis, newborns

## Abstract

**Highlights:**

**What are the main findings?**

• Staphylococcus aureus neonatal osteomyelitis is a condition that primarily affects full-term male neonates, typically involving long bones such as the femur, humerus, and tibia.
• It presents with local swelling and reduced mobility, requiring prolonged intravenous antibiotic treatment for successful resolution.

**What is the implication of the main finding?**

• Immediate diagnosis is essential to limit damage to rapidly growing neonatal bones; however, the risk of long-term sequelae should not be underestimated.
• Given the heterogeneity of current available scientific data, rigorous and standardized pediatric follow-up is necessary to monitor clinical evolution over time.

**Abstract:**

**Background:** *Staphylococcus aureus* neonatal osteomyelitis (SA-NOm) is a rare condition with the potential for lifelong skeletal morbidity. Available evidence remains scarce and inconsistent, with notable differences in clinical presentation, therapeutic regimens, and reported outcomes, underscoring the need for a systematic evaluation combining clinical experience with existing literature. **Methods:** We retrospectively reviewed data from all neonates admitted to Regina Margherita Children’s Hospital, Turin, Italy, between 2017 and 2024 with a diagnosis of SA-NOm. A structured narrative review of the pertinent literature published over the past 25 years was conducted to identify additional cases and compare management approaches. **Results:** Four neonates with SA-NOm were identified at our center (institutional cohort) while a literature review retrieved 38 additional cases (literature cohort) to establish a combined cohort (n = 42). Of these, 78% were born at term, with a male-to-female ratio of 1.6:1 (26 males, 16 females). Approximately half of the combined cohort presented identifiable risk factors for SA-NOm, including neonatal intensive care unit admission, prematurity, sepsis, or maternal complications. Across the combined cohort, the mean age at presentation was 19 days. The most common presenting signs were local swelling and reduced mobility of the affected limb, although systemic symptoms often complicated early recognition. Long bones were most frequently involved—particularly the femur, humerus, and tibia—with equal distribution between upper and lower extremities. The mean intravenous antibiotic duration for the combined cohort was 31.6 days, followed by two to three weeks of oral therapy. Empiric regimens varied, including glycopeptides alone or combined with second- or third-generation cephalosporins, anti-staphylococcal penicillins, or carbapenems. Sequelae rates were rarely reported in the literature, likely due to limited follow-up, whereas extended surveillance in our cohort revealed substantial long-term morbidity, including restricted joint mobility, limb length discrepancy, and persistent radiographic abnormalities. **Conclusions:** SA-NOm, due to its rarity and potential for long-term skeletal sequelae, requires early diagnosis and timely empiric antibiotic therapy based on local resistance data. Prospective multicenter studies are needed to define standardized diagnostic and therapeutic protocols.

## 1. Introduction

Neonatal osteomyelitis (NOm) is a rare but serious bone infection, often associated with significant morbidity and mortality. Recent epidemiological data [1] indicate an incidence of one to seven cases per 1000 hospital admissions, consistent with earlier reports [2,3,4] but substantially higher than the incidence observed in the overall pediatric population [5,6,7]. However, precise epidemiological figures remain elusive due to the lack of large-scale, standardized studies; therefore, any definitive estimation of the true incidence at this stage would be premature and potentially inaccurate.

Hematogenous spread is the predominant route of infection in neonates, and *Staphylococcus aureus* (SA) remains one of the most frequently isolated pathogens. Despite the potentially lifelong morbidity associated with SA-related neonatal osteomyelitis (SA-NOm), few studies have specifically focused on its clinical features and management, and published data show wide variability in case descriptions and conclusions. Therefore, this study aims to describe the clinical presentation and therapeutic management of SA-NOm through a combined case series and structured narrative review.

## 2. Materials and Methods

We retrospectively reviewed all neonates with SA-NOm admitted to the Regina Margherita Children’s Hospital (Turin, Italy) between January 2017 and December 2024. Four patients met the predefined eligibility criteria, establishing our institutional cohort (n = 4). Their clinical, radiological and microbiological data were extracted from medical records and evaluated alongside 38 cases identified in the literature over the past 25 years.

Eligibility was determined by the following inclusion criteria: (1) radiological evidence of osteomyelitis on plain radiography, computed tomography (CT), or magnetic resonance imaging (MRI); (2) microbiological isolation of SA from blood cultures, bone aspirates, or synovial fluid; and (3) symptom onset at ≤30 days of life for term neonates, or diagnosis by 40 weeks corrected gestational age for preterm infants. Exclusion criteria included: (1) insufficient clinical, radiological, or microbiological data precluding comprehensive case characterization; (2) osteomyelitis attributable to pathogens other than SA; and (3) symptom onset exceeding the predefined age limits.

A structured narrative review of the literature from January 2000 to December 2024 was conducted across PubMed/MEDLINE, Embase, Web of Science, and Google Scholar. The search strategy combined the following terms using Boolean operators: (“osteomyelitis” OR “osteoarthritis”) AND (“neonate” OR “newborn” OR “infant”) AND (“*Staphylococcus aureus*” OR “*S. aureus*”). The conventional search was supplemented by System Pro (System.com), an artificial intelligence (AI) tool for semantic searching and citation network analysis, to retrieve older or non-standardized reports that might be overlooked by standard keyword queries. To preserve methodological rigor and limit automated selection bias, the AI served solely as an initial discovery aid; all identified records were manually validated by the authors to determine final eligibility. Additionally, reference lists of retrieved articles were screened for further relevant studies. Overall, the literature search yielded 18 publications detailing 38 cases (literature cohort, n = 38). These were pooled with our four institutional patients (institutional cohort, n = 4), establishing a final study population of 42 neonates (combined cohort, n = 42). Consistent with the narrative review design, formal comparative statistics were not performed, and data were summarized purely via descriptive analysis.

## 3. Results

We included four institutional patients (Table 1) and 38 cases retrieved from the literature (Table 2).

### 3.1. Clinical Profile and Risk Factors

Data analysis revealed a male predominance and a mean age at symptom onset of 18.5 days, consistent with the literature. Predisposing factors were identified in all institutional patients (100%) compared with 45% of the literature cases; this discrepancy likely reflects inconsistent documentation in retrospective studies. Among the reviewed literature, NICU admission (23%), prematurity (18%), and systemic sepsis (16%) were the most frequently reported conditions. Conversely, our series demonstrated higher rates of prematurity (50%, 2/4) and sepsis (75%, 3/4). Maternal complications (e.g., infections, bleeding, preeclampsia, and placental abruption) were noted in 13% of published cases versus 25% (1/4) locally. Immunodeficiencies and localized skin infections constituted additional risk factors across both groups. Clinically, 70% of the literature cohort exhibited localized inflammatory signs (e.g., swelling, reduced limb mobility), whereas such focal findings were less prominent in our patients. Furthermore, fever was documented in only 31% of literature cases, compared with 75% (3/4) of our patients (Table 3).

### 3.2. Anatomical Distribution

**Figure 1:**
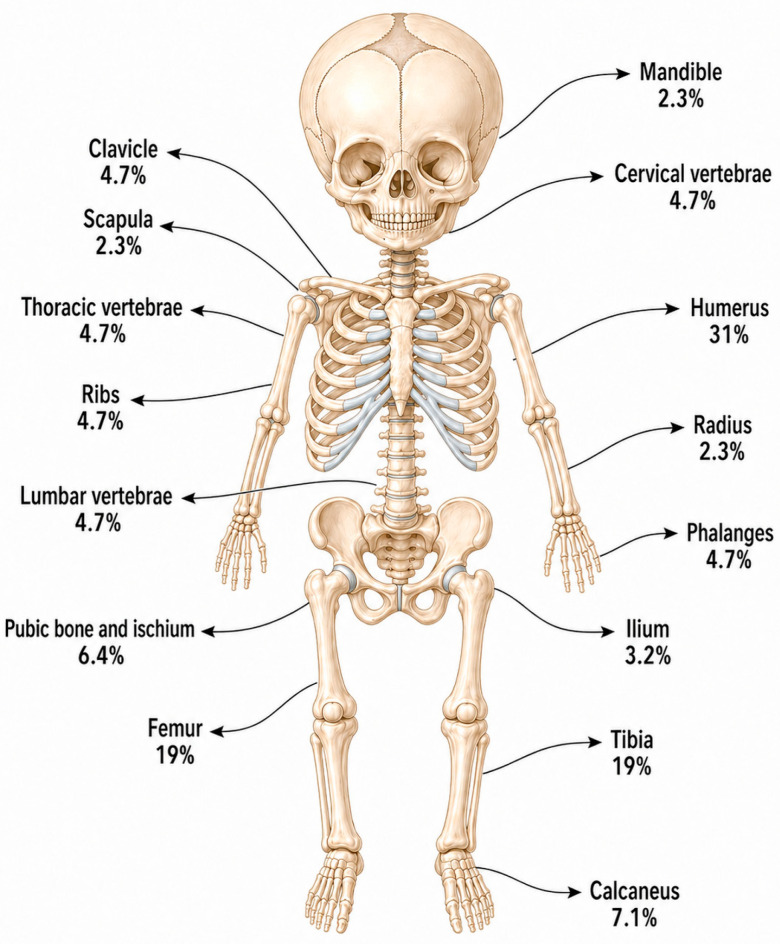
Involved sites of SA-NOm considering the combined cohort (n = 42).

The anatomical distribution of SA-NOm in the combined cohort (n = 42) is detailed in Figure 1. Subgroup analysis highlighted single-site infection as the predominant pattern in both the literature (71.1%) and institutional (100%, 4/4) groups. Regarding clinical presentation, isolated bone lesions predominated in the literature cohort (86.8%), whereas combined osteoarticular extension was less frequent (13.2%, 5/38) compared to our institutional series (25%, 1/4). Regionally, the upper extremities were the most frequently affected sites. Humeral infections were reported in 28.9% of literature cases versus 50% (2/4) of our patients, with shoulder joint extension observed in 7.9% and 25%, respectively. Lower extremity involvement affected 36.8% of the reviewed neonates; similarly, one institutional patient exhibited isolated femoral disease (25%, 1/4). Additionally, a rare costal (rib) localization was noted in our cohort (25%, 1/4).

### 3.3. Microbiology and Diagnostic Workup

Bacteremia was confirmed in all patients of the institutional cohort (100%), aligning with rates exceeding 80% in the literature cohort. Data on extraosseous complications were sparse; infective endocarditis, pulmonary abscesses, and meningitis were not systematically described in the reviewed reports. In the institutional cohort, a comprehensive diagnostic workup was implemented. Echocardiography yielded no evidence of cardiac anomalies. Additional systemic evaluations included abdominal and cranial ultrasounds, and targeted imaging when clinically indicated (50%, 2/4). To definitively exclude central nervous system infection prior to antibiotic de-escalation, a brain MRI was obtained for two neonates (50%, 2/4) exhibiting mild ventricular hyperechogenicity. Chest radiography was conducted in two cases, and the patient with the rib lesion additionally underwent a chest CT scan. Finally, an immunological workup (serum immunoglobulin levels and lymphocyte subset analysis) was completed for the entire institutional cohort.

### 3.4. Laboratory and Microbiological Findings

In both cohorts, initial laboratory evaluations demonstrated elevated white blood cell (WBC) counts (median: 21,100 vs. 22,900 cells/µL, institutional vs. literature) and C-reactive protein (CRP) levels (median: 93 vs. 82 mg/L). SA was isolated in all cases; however, the specific sampling site was not specified in 23.7% (9/38) of the literature cohort. In the institutional cohort, exclusively methicillin-susceptible SA (MSSA) was isolated. Conversely, methicillin-resistant SA (MRSA) strains accounted for 39.5% (15/38) of the published reports. Further classification of these strains as healthcare-associated (HA-MRSA) or community-acquired (CA-MRSA) was precluded by a lack of comprehensive clinical data, antibiograms, and molecular characterization in the reviewed studies. Additionally, data regarding molecular typing or toxin production were absent in the literature, whereas Panton-Valentine leukocidin (PVL) testing was uniformly negative across our institutional cohort.

### 3.5. Radiological Imaging

At the onset of NOm, imaging modalities varied significantly in their diagnostic yield. In the literature cohort, pathological findings were identified on initial radiography (X-ray) in 44% of cases, magnetic resonance imaging (MRI) in 34.2%, ultrasound (US) in 13%, and computed tomography (CT) in 13%. In contrast, within the institutional cohort, initial X-rays revealed pathological changes in only 25% (1/4) of patients, whereas both MRI and US successfully identified abnormalities in 75% (3/4). Morphologically, common radiographic signs included periosteal reaction and bone lysis, while MRIs typically demonstrated subperiosteal abscesses with adjacent soft tissue involvement. Notably, initial evaluations utilizing X-rays and ultrasounds frequently yielded false-negative results across the combined cohort (Table 4).

### 3.6. Treatment and Surgical Management

Treatment comprised both medical and surgical approaches. In the institutional cohort, given a local MRSA prevalence exceeding 10%, all patients received empirical anti-MRSA therapy with a glycopeptide. This was combined, as clinically indicated, with aminoglycosides, cephalosporins, or antistaphylococcal penicillins in accordance with current guidelines. Once microbiological susceptibility results became available, therapy was de-escalated to a first-generation cephalosporin (cefazolin). Targeted adjunctive therapy was individualized based on clinical severity and pathogen characteristics: clindamycin was added in one case due to suspected PVL-producing MSSA (subsequently discontinued after negative confirmation), linezolid in a second case, and daptomycin in a third. Intravenous therapy was maintained for four weeks, followed by three weeks of oral treatment. Surgical intervention was required in two of the four patients.

In the literature cohort, all patients received prolonged antibiotic therapy (≥6 weeks), and 60% (23/38) also underwent surgery. The mean duration of intravenous treatment was 32 days, followed by 2–3 weeks of oral therapy. Glycopeptides (specifically vancomycin, and rarely teicoplanin) were the most frequently administered agents (63%), followed by cephalosporins (34%) and antistaphylococcal penicillins (24%). Vancomycin was typically used alone or in combination with a cephalosporin, aminoglycoside, or carbapenem to ensure broad-spectrum coverage. Antistaphylococcal penicillins (e.g., oxacillin, cloxacillin) combined with aminoglycosides (frequently gentamicin or netilmicin), were preferred in cases of neonatal sepsis, whereas cephalosporins or penicillins as monotherapy were limited to settings with low MRSA prevalence. Oral step-down agents included amoxicillin–clavulanate, clindamycin, and linezolid. Surgical intervention—primarily incision and drainage—was performed in 23 cases, with two patients requiring repeated procedures (Table 5).

### 3.7. Follow-Up and Clinical Outcomes

All patients in the institutional cohort completed follow-up (mean: 24.8 months; range: 19–28). Sequelae were observed in 75% (3/4) of these cases: those with shoulder involvement demonstrated a reduced range of motion, mild upper limb hypotrophy, and persistent radiological changes in the proximal humerus. The patient with femoral involvement experienced no sequelae, whereas rib involvement was associated solely with minor radiological findings. In the literature cohort, sequelae were reported in 26.3% (10/38) of cases—predominantly limb length discrepancy and joint mobility limitations—although long-term outcome data were unavailable for nearly half of the published reports.

## 4. Discussion

NOm is a rare condition, and the incidence of SA-related cases remains poorly defined [8,9]. The absence of uniform diagnostic criteria and significant heterogeneity in reporting hinder the establishment of a consistent clinical profile.

Recent data suggest a male predominance in SA-NOm [10], a pattern reflected in the present cohort. Although our initial M:F ratio was slightly higher than the previously described 1.5:1—likely due to the small sample size—pooled analysis normalized this ratio to 1.6:1. Furthermore, the mean age at symptom onset (~19 days) was consistent with published data, suggesting a relatively homogeneous clinical presentation for this rare infection. Predisposing conditions were documented in only 45% of reviewed cases, whereas they were present in all patients in our series. This discrepancy likely reflects underreporting in the literature rather than true epidemiological differences, as nearly half of the published cases did not specify risk factors. Despite this reporting heterogeneity, the principal risk factors for SA-NOm remained consistent across studies: NICU admission, prematurity, and sepsis emerged as the dominant predisposing factors in both the literature and our cohort, with a higher prevalence observed in our series. NICU hospitalization increases exposure to invasive procedures and predisposes neonates to nosocomial infections, often caused by multidrug-resistant organisms [11]. In the present series, two patients developed MRSA infections during their NICU stay, consistent with findings by Roversi et al. [11]. Additionally, three out of four patients were born preterm, reinforcing immune immaturity as a critical predisposing factor [12]. Maternal complications—documented in 17% of literature cases and in one patient in our series—also appear to play a role in increasing the risk of SA-NOm [9].

NOm frequently presents with non-specific symptoms, complicating early diagnosis. The majority of reviewed cases exhibited local inflammatory signs, such as swelling and reduced limb mobility, while fever was notably less common [11]. In contrast, only one out of four patients in our series displayed local signs; instead, the majority presented with fever, suggesting a predominantly systemic clinical presentation. This divergence may reflect the higher prevalence of prematurity and concurrent sepsis observed in our cohort, which may mask localized findings in favor of generalized illness. Furthermore, this difference likely highlights the impact of modern Neonatal Intensive Care Unit (NICU) monitoring. Continuous clinical surveillance allows for the prompt detection of early systemic inflammatory responses during the initial bacteremic phase, often intercepting the infection before classic focal symptoms, such as soft-tissue swelling or pseudo-paralysis, fully manifest.

The distribution of SA-NOm reflects neonatal vascular anatomy, where persistent transphyseal vessels facilitate the hematogenous spread of infection to the epiphysis and adjacent joints [13]. While long bones—particularly those of the lower extremities such as the femur and tibia—are traditionally reported as the most frequent sites of involvement in both paediatric and neonatal osteomyelitis [14], our findings indicate that both upper and lower limbs are equally involved, with the humerus, femur, and tibia being the most affected [12,15]. Osteoarticular extension was notably more frequent in our series compared to the reviewed literature (25% vs. 7.9%). Although this discrepancy may be influenced by our small sample size, it remains consistent with the mechanism of transphyseal spread and underscores the importance of differentiating isolated osteomyelitis from joint involvement to guide surgical planning. Furthermore, the rich vascular supply of the developing axial skeleton facilitates haematogenous seeding similar to appendicular sites; our observation of rib involvement reinforces the need for vigilance regarding atypical localisations in neonates with SA [15]. Finally, cutaneous colonisation by SA may predispose neonates to infection at sites of invasive procedures, as illustrated by cases of calcaneal osteomyelitis following Guthrie card screening or vascular access [16,17].

Laboratory findings typically reveal elevated WBC and CRP levels, although ESR is rarely reported in the literature. While essential, conventional X-rays may yield false negatives if performed too early [10]. In the reviewed cases, X-rays were the most frequently used imaging modality, yet a significant proportion showed no initial abnormalities. Although ultrasound proved useful in detecting soft-tissue abscesses, it remained non-definitive for bone infection. In contrast, MRI and CT consistently identified pathological changes, supporting their pivotal role in early and precise diagnosis. Microbiological confirmation of SA was achieved in most cases through blood, synovial fluid, or bone aspirates.

SA-NOm should be approached as a systemic disease rather than a localised bone infection. However, data on extra-osseous complications remain limited in the reviewed literature. Neonatal SA infective endocarditis (IE), increasingly reported in NICU infants with central venous catheters, may lead to septic emboli and metastatic dissemination [18]. In our series, we routinely performed echocardiography in all patients to exclude cardiac involvement, consistent with current recommendations for SA bacteremia current recommendations [19]. While whole-body MRI has been described as a valuable tool for identifying multifocal involvement in pediatric osteomyelitis, its application is currently guided by clinical suspicion rather than formal protocols [19]. Prospective studies are essential to establish standardized screening for disseminated neonatal SA-NOm.

Notably, MRSA strains accounted for over one-third of reported cases, reinforcing the need for adequate empirical coverage [8]. This global prevalence contrasts with our institutional cohort, which consisted exclusively of MSSA cases, despite regional epidemiological data indicating otherwise. This discrepancy likely reflects the low-risk profile of our population, as the mothers had unremarkable clinical histories, with no prior hospitalizations or antibiotic exposures typically associated with resistant strains. Consistent with these findings, maternal MRSA screenings in our series were uniformly negative. However, this does not definitively preclude the vertical transmission of MSSA or early horizontal acquisition. Currently, there is insufficient data regarding the classification of these isolates as HA- or CA-MRSA (healthcare- or community-associated). The reviewed literature lacks comprehensive microbiological resistance studies, which are crucial for optimizing therapeutic strategies—especially given SA’s ability to genetically adapt to conventional regimens [20]. Furthermore, data on PVL-producing strains remains scarce, despite their known association with aggressive disease and treatment resistance. Since PVL can be produced by both MSSA and MRSA, screening for toxin-mediated pathology is vital for treatment planning [21]. In our series, PVL testing was systematically performed and consistently yielded negative results.

In neonatal osteomyelitis, empiric antibiotic therapy must provide broad-spectrum coverage against the primary pathogens involved, particularly for neonates at high risk for nosocomial infections. The choice of the initial empirical antibiotic regimen depends on patient history, clinical severity, local MRSA prevalence, and clinical judgement. According to current pediatric guidelines [10,22], in regions with low MRSA prevalence (less than 10–15%), narrow-spectrum agents such as oxacillin, nafcillin, or cefazolin are recommended for children with mild to moderate illness, provided there is close clinical monitoring. Conversely, in regions with high MRSA prevalence (>15%), empiric coverage should include anti-MRSA agents, typically clindamycin or vancomycin. Regardless of local resistance patterns, vancomycin remains the standard initial choice for any child presenting with severe or life-threatening symptoms. Notably, these recommendations are tailored for the general pediatric population; while they provide a strategic framework, they do not address the specific therapeutic nuances or adjusted dosages required for the neonatal period.

Within the reviewed literature on SA-NOm, vancomycin-based combinations emerge as the most widely adopted empirical approach. These protocols are frequently associated with aminoglycosides or third-generation cephalosporins to ensure broad-spectrum coverage during the initial phase of treatment, prior to pathogen identification. Targeted therapy is subsequently adjusted based on microbiological isolates, when available, and clinical response: vancomycin remains the cornerstone for MRSA, whereas de-escalation to narrow-spectrum beta-lactams is indicated for MSSA, in line with pediatric guidelines [10,22]. However, data regarding de-escalation practices for MSSA cases in the reviewed studies remain insufficient. In our cohort, anti-MRSA empiric therapy was initiated as first-line treatment due to a 10% prevalence, followed by de-escalation at 72 h once microbiological results were available, consistent with antimicrobial stewardship principles. The median duration of intravenous therapy was 32 days, followed by two or three weeks of oral treatment. The literature lacks definitive data regarding the optimal timing for the intravenous-to-oral switch; consequently, treatment duration and administration route remain subjects of debate. Current guidelines suggest that while parenteral therapy is typically required for 3 to 4 weeks in uncomplicated infections, an extended course of 4 to 6 weeks may be necessary for complicated cases. These include infections involving MRSA or PVL+ strains, newborns and young infants, cases with a slow clinical response, or involvement of the pelvis and spinal column [10]. A transition to oral therapy may be considered after clinical stabilization and a decrease in inflammatory markers, with a total course spanning 3 to 6 weeks, depending on clinical evolution [10]. Furthermore, evidence concerning newer antibiotics, such as linezolid or daptomycin, remains extremely limited in the current literature [12,23,24]. In our experience, patients received a total of six weeks of therapy (intravenous followed by oral). Ultimately, treatment duration should be tailored based on local resistance patterns, clinical response, and biomarker trends [23]. Finally, the role of surgery remains debated; while early antibiotic therapy is prioritized [10], aggressive cases—especially those involving PVL-positive strains—may require repeated surgical interventions [23].

Surgical intervention may enhance antibiotic penetration, reduce bacterial load, and lower the risk of long-term sequelae [15]. The 2017 European Society for Paediatric Infectious Diseases guidelines for bone and joint infection recommend considering surgery in children if there is no clinical response to antibiotic therapy within a few days or if a complication is suspected [10]. Similarly, the 2021 American guidelines on the management of acute pediatric osteomyelitis strongly recommend immediate surgical debridement of infected bone and associated abscesses (particularly those >2 cm) for stable children not responding to therapy or patients presenting with sepsis and rapidly progressive infections, rather than medical therapy alone [22]. Regarding NOm, however, there is limited evidence from randomized controlled trials to guide surgical management. In the reviewed literature, approximately half of the cases underwent surgery, a finding consistent with our cohort. Despite these observations, critical questions regarding the optimal timing, extent, and necessity of surgery—beyond its role in obtaining diagnostic biopsies—remain unresolved in the neonatal population.

Follow-up data in the reviewed literature remain limited. In our series, three patients were followed for more than 24 months to monitor radiological resolution and motor function. However, established guidelines suggest that infants should be monitored by experienced orthopedic surgeons and pediatricians for a variable duration, depending on infection severity, age, and the affected site. This surveillance must include a comprehensive assessment of clinical status, laboratory markers, and imaging [10]. Clinical monitoring should encompass a multidisciplinary evaluation of functional impairment (e.g., restricted joint range of motion), skeletal growth disturbances (including physeal arrest and limb length discrepancy), and radiographic evidence of structural alterations, such as cortical hypertrophy or bone remodeling. Long-term follow-up is critical in NOm, as bone development during childhood is a highly dynamic and accelerated process, with peak growth velocities occurring from birth to age two and again during puberty. During follow-up, two of our cases exhibited reduced mobility of the affected limb, and one patient presented with limb length discrepancy. Radiologically, three cases showed persistent alterations of the bone profile. In the reviewed literature, limited information was provided regarding the sequelae of the patients involved; the few mentioned included motor disability, limb length discrepancy, and reduced range of motion.

This study has several strengths. Despite the rarity of SA-NOm, our analysis of microbiologically confirmed cases over 25 years provides valuable clinical insights. It identifies age-specific clinical patterns and supports the optimization of diagnostic and therapeutic strategies for neonatologists and paediatric infectious disease specialists. Furthermore, evaluating both clinical and radiological sequelae over a comprehensive 24-month period adds significant longitudinal value to the current evidence base. Crucially, this extended observation strongly suggests that prior reports underestimate true long-term morbidity due to limited longitudinal follow-up. However, certain limitations must be acknowledged. The retrospective design and the small sample size (n = 4) represent inherent limitations of this study. Moreover, the existing literature is limited by inconsistent reporting of antimicrobial transition criteria, variable follow-up durations, and a lack of standardized diagnostic definitions. Collectively, these factors underscore the urgent need for prospective, multicenter data to establish evidence-based guidelines for the clinical management of this rare and complex condition.

## 5. Conclusions

SA-NOm is a rare yet severe infection requiring prompt recognition and timely management. NICU admission, prematurity, and invasive intravascular access have been identified as age-specific risk factors. The high frequency of humeral involvement represents a key diagnostic “red flag” during clinical evaluation. Empirical antibiotic therapy should be tailored to local epidemiology; furthermore, the presence of resistant strains or PVL-toxin should be suspected in cases of treatment failure, extensive tissue involvement, or significant bone destruction requiring surgical intervention. Given the risk of systemic complications, particularly IE, the proactive use of echocardiography in SA-NOm cases is incorporated into our routine clinical practice to ensure comprehensive and early assessment. Finally, due to the rarity of this condition, prospective multicenter studies utilizing standardized protocols are essential to better characterize its clinical course and optimize management.

## Figures and Tables

**Table 1:** Clinical findings in institutional cohort.

	Risk Factor	Clinical Presentation	WBCs/µL at Onset	CRP mg/L at Onset	Bacteriology	Imaging	Bones	IV Antibiotics *(Days)* + Oral Antibiotics *(Days)* Surgery	Follow-Up *(Months)* Outcome *(Clinical/Radiological)*
Case 1	Late prematurity Maternal Ureaplasm infection	Limb swelling Pain	16.450	69	Pus culture PLV-negative MSSA Blood culture-MSSA	X-rays US MRI	Humerus	IV cefazolin and linezolid (28) + oral linezolid (14) Debridement	26 Clinical: reduced active shoulder flexion above 90°, mild asymmetry of the shoulders, slight hypotrophy of the left arm Radiological: architectural bone alterations of the proximal humerus
Case 2	Newborn Sepsis Peripheral IV site infection	Fever Irritability Poor feeding	30.740	230	Pus culture PLV-negative MSSA Blood culture-MSSA	X-rays US CT scan	Rib	IV ampicillin/sulbactam, teicoplanin and gentamicin, then cefazolin (28) + oral amoxicillin/clavulanic acid (14) Debridement	26 Clinical/radiological: none
Case 3	Newborn Sepsis	Fever Irritability	19.100	10.7	Blood culture PLV-negative MSSA	US X-rays MRI	Femur	IV ampicillin and netilmicin, then cefazolin and clindamycin (28) + oral amoxicillin/clavulanic acid (14) No	28 Clinical: none Radiological: mild irregularity of the lesser trochanter
Case 4	Prematurity Sepsis	Fever Irritability Poor feeding Respiratory distress	25.360	97	Blood culture PLV-negative MSSA	X-rays US MRI	Humerus	IV cefazolin and daptomycin (28) + oral amoxicillin/clavulanic acid (56) No	19 Clinical: mild limitation of active left shoulder elevation. Radiological: persistent architectural bone alterations of the proximal humeral metaphysis, bone remodelling and early structural changes in the humeral head

**Table 2:** Cases in literature from the past 25 years.

Title	Authors	Journal	Year	N° of Cases
Neonatal osteomyelitis: an Italian multicentre report of 22 cases and comparison with the inherent literature	*Roversi, M. et al.*	Journal of Perinatology	2021	4 *
Osteomyelitis in immunocompromised children and neonates: a case series	*Foong, B. et al.*	BMC Paediatrics	2021	2
Osteomyelitis in the neonate	*McPherson, D.M.*	Neonatal Network	2002	1
Methicillin-resistant *Staphylococcus aureus* osteomyelitis and septic arthritis in neonates: diagnosis and management	*Korakaki, E. et al.*	Journal of Infectious Diseases	2007	2
Neonatal cervical osteomyelitis with bilateral upper limb paresis	*Ben Meir, E. et al.*	The Paediatric Infectious Disease Journal	2017	1
Chronic osteomyelitis of clavicle in a neonate: report of morbid complication of adjoining MRSA abscess	*Suranigi, S.M. et al.*	Case Reports in Pediatrics	2016	1
Calcaneus osteomyelitis secondary to Guthrie test: case report	*Tural Kara, T. et al.*	Arch Argent. Pediatric	2016	1
Hypogammaglobulinemia causing multiple abscesses and osteomyelitis of calcaneus following a heel puncture in a preterm neonate	*Deora, K. et al.*	Cureus	2023	1
Treatment of persistent methicillin-susceptible *Staphylococcus aureus* bacteremia and presumed osteomyelitis with oxacillin and ertapenem in a premature neonate	*Hitchins, M. et al.*	Pharmacotherapy	2023	1
Methicillin-resistant *Staphylococcus aureus* mandibular osteomyelitis in an extremely low birth weight preterm infant	*Martini, S. et al.*	Italian Journal of Pediatrics	2015	1
Immunological profile of neonatal osteomyelitis cases (2002–2019)	*Sako, I. et al.*	Clinical Case Reports	2021	2
Clinical analysis of 17 cases of neonatal osteomyelitis	*Zhan, C. et al.*	Medicine	2019	10
Bone and joint infection complicated with sepsis in neonates and infants under three months of age	*Liu, Y. et al.*	Jornal de Pediatria	2023	5
Osteomyelitis of the calcaneus in the new-born: an ongoing complication of Guthrie test	*Yuksel, S. et al.*	European Journal of Pediatrics	2007	1
Neonatal cervical osteomyelitis with paraspinal abscess and Erb’s palsy: a case report and brief review of the literature	*Sharma, R.R. et al.*	Pediatric Neurosurgery	2000	1
Multifocal neonatal osteomyelitis: a case report	*Manisha, A. et al.*	Scholars Journal of Medical Case Reports	2022	1
Newborn with primary sternal osteomyelitis and systemic sepsis caused by X-linked Hereditary Congenital Neutropenia: a case report and literature review	*Khalil, A.H. et al.*	World Journal of Surgery and Surgical Research	2023	1
Neonatal osteomyelitis: a case series	*Kumar, P. et al.*	Journal of Neonatal Surgery	2023	2

* Out of the 22 neonatal osteomyelitis cases reported by Roversi, M. et al., only 4 cases were explicitly identified as being caused by *Staphylococcus aureus* with available individual-level clinical data.

**Table 3:** Clinical characteristics of patients.

	Institutional Cohort	Literature Cohort
Number of cases	4	38
Age (days)	18.5	19
Sex (M:F)	3 M 1 F (3.0:1)	23 M 15 F (1.5:1)
Risk factors		
Yes (*n*, %)	4 (100%)	17 (44.7%)
Admission in NICU (*n*, %)		9 (23.7%)
Sepsis (*n*, %)	3 (75%)	6 (15.8%)
Prematurity (*n*, %)	2 (50%)	7 (18.4%)
Maternal Complications (*n*, %)	1 (25%)	5 (13.2%)
Immunodeficiency (*n*, %)		2 (5.3%)
Others * (*n*, %)		10 (26.3%)
No (*n*, %)	1 (25%)	5 (13.2%)
Not specified (*n*, %)		16 (42.1%)
Fever		
Yes (*n*, %)	3 (75%)	12 (31.6%)
No (*n*, %)	1 (25%)	12 (31.6%)
Not specified (*n*, %)		14 (36.8%)
Local symptoms (*n*, %)	1 (25%)	27 (71.1%)
Not specified (*n*, %)		6 (15.8%)
Sites		
Upper limb (*n*, %)	2 (50%)	16 (42.2%)
Lower limb (*n*, %)	1 (25%)	14 (36.8%)
Axial/craniofacial (*n*, %)	1 (25%)	8 (21%)
Isolated osteomyelitis (*n*, %)	3 (75%)	33 (86.8%)
Osteoarticular infection (*n*, %)	1 (25%)	5 (13.2%)

M: Male; F: Female; NICU: Neonatal Intensive Care Unit. * Haematological disorders (AB0 incompatibility, intracranial haemorrhage, anaemia, haemolysis), osteopetrosis, metabolic screening with Guthrie card, cardiac and respiratory illness, peripheral line cannulation.

**Table 4:** Laboratory examination, bacteriology and imaging studies.

	Institutional Cohort	Literature Cohort
Number of cases	4	38
Laboratory test		
WBC median value cells/µL *(IQR)*	22,900 (17,775–28,050)	21,100 (14,565–27,625)
Not specified (*n*)		17
CRP median value mg/L *(IQR)*	82 (39.85–163.5)	93 (39.95–127.55)
Increased >5 mg/L (*n*)	4	20
Normal (*n*)		1
Not specified (*n*)		17
Microbiology		
Synovial fluid/bone sample (*n*, %)	2 (50%)	21 (55.3%)
Blood (*n*, %)	4 (100%)	20 (52.6%)
Not specified (*n*, %)		9 (23.7%)
Type of SA strain		
MSSA (*n*, %)	4 (100%)	23 (60.5%)
MRSA (*n*, %)		15 (39.5%)
Imaging at presentation		
X-rays pathologic (*n*, %)	1 (25%)	17 (44.7%)
Negative (*n*, %)		9 (23.7%)
US pathologic (*n*, %)	3 (75%)	5 (13.2%)
Negative (*n*, %)		11 (28.9%)
MRI pathologic (*n*, %)	3 (75%)	13 (34.2%)
Negative (*n*, %)		0 (0%)
CT scan pathologic (*n*, %)	1 (25%)	5 (13.2%)
Negative (*n*, %)		0 (0%)
Not specified (*n*, %)		8 (21.1%)

SA: *Staphylococcus aureus*; US: Ultrasound; MRI: Magnetic resonance imaging; CT: Computed tomography.

**Table 5:** Management.

	Institutional Cohort	Literature Cohort
Number of cases	4	38
Surgery		
Yes (n, %)	2 (50%)	23 (60.5%)
Not specified (n, %)		8 (21.1%)
Antibiotics		
IV		
Time days *(IQR)*	28 (28–28)	32 (21–42)
Types of antibiotics (*n*, %)		
Penicillin	2 (50%)	9 (23.7%)
Glycopeptides	1 (25%)	24 (63.2%)
Teicoplanin	*1* (*25*%)	*1 *(*2.6*%)
Vancomycin		*23 *(*60.5*%)
Cephalosporin	4 (100%—I° gen.)	13 * (34.2%)
Aminoglycosides	2 (50%)	8 (21.1%)
Gentamicin	*1 (25%)*	*6 (15.8%)*
Netilmicin	*1 *(*25*%)	*2 (5.3%)*
Clindamycin	1 (25%)	1 (2.6%)
Carbapenem		6 (15.8%)
Linezolid	1 (25%)	2 (5.3%)
Daptomycin	1 (25%)	1 (2.6%)
ORAL		
Time days *(IQR)*	17.5 (14–35)	20 (14–30)
Not specified (*n*, %)		25 (65.8%)

* (4—I° gen, 1—II° gen, 8—III° gen).

## Data Availability

The data presented in this study are available on request from the corresponding author due to privacy and ethical restrictions.

## Abbreviations

The following abbreviations are used in this review:

SA-NOm	*Staphylococcus aureus* neonatal osteomyelitis
NOm	Neonatal osteomyelitis
NICU	Neonatal intensive care unit
WBC	White blood cell count
SA	*Staphylococcus aureus*
MRSA	Methicillin-resistant *Staphylococcus aureus*
MRI	Magnetic resonance imaging
CT	Computed tomography
CRP	C-reactive protein
ESR	Erythrocyte sedimentation rate
PVL	Panton–Valentine leukocidin
MSSA	Methicillin-susceptible *Staphylococcus aureus*
ESPID	European society for pediatric infectious disease
AI	Artificial intelligence
IE	Infective endocarditis
CA-MRSA	Community-acquired MRSA
HA-MRSA	Healthcare-acquired MRSA
